# Closing the gap: did delivery approaches complementary to home‐based testing reach men with HIV testing services during and after the HPTN 071 (PopART) trial in Zambia?

**DOI:** 10.1002/jia2.25855

**Published:** 2022-01-09

**Authors:** Mwelwa Muleba Phiri, Ab Schaap, Musonda Simwinga, Bernadette Hensen, Sian Floyd, Chama Mulubwa, Melvin Simuyaba, Bwalya Chiti, Virginia Bond, Kwame Shanaube, Sarah Fidler, Richard Hayes, Helen Ayles, the HPTN 071 (PopART) Study team

**Affiliations:** ^1^ Zambart Lusaka Zambia; ^2^ Department of Infectious Disease Epidemiology London School of Hygiene and Tropical Medicine London UK; ^3^ Clinical Research Department London School of Hygiene and Tropical Medicine London UK; ^4^ Department of Global Health London School of Hygiene and Tropical Medicine London UK; ^5^ Imperial College and Imperial College NIHR BRC London UK

**Keywords:** men, HIV testing, Zambia, sub‐Saharan Africa, community‐based

## Abstract

**Introduction:**

The HPTN 071 (PopART) trial demonstrated that universal HIV testing‐and‐treatment reduced community‐level HIV incidence. Door‐to‐door delivery of HIV testing services (HTS) was one of the main components of the intervention. From an early stage, men were less likely to know their HIV status than women, primarily because they were not home during service delivery. To reach more men, different strategies were implemented during the trial. We present the relative contribution of these strategies to coverage of HTS and the impact of community hubs implemented after completion of the trial among men.

**Methods:**

Between 2013 and 2017, three intervention rounds (IRs) of door‐to‐door HTS delivery were conducted in eight PopART communities in Zambia. Additional strategies implemented in parallel, included: community‐wide “Man‐up” campaigns (IR1), smaller HTS campaigns at work/social places (IR2) and revisits to households with the option of HIV self‐testing (HIVST) (IR3). In 2018, community “hubs” offering HTS were implemented for 7 months in all eight communities. Population enumeration data for each round of HTS provided the denominator, allowing for calculation of the proportion of men tested as a result of each strategy during different time periods.

**Results:**

By the end of the three IRs, 65–75% of men were reached with HTS, primarily through door‐to‐door service delivery. In IR1 and IR2, “Man‐up” and work/social place campaigns accounted for ∼1 percentage point each and in IR3, revisits with the option of self‐testing for ∼15 percentage points of this total coverage per IR. The yield of newly diagnosed HIV‐positive men ranged from 2.2% for HIVST revisits to 9.9% in work/social places. At community hubs, the majority of visitors accepting services were men (62.8%). In total, we estimated that ∼36% (2.2% tested HIV positive) of men resident but not found at their household during IR3 of PopART accessed HTS provided at the hubs after trial completion.

**Conclusions:**

Achieving high coverage of HTS among men requires universal, home‐based service delivery combined with an option of HIVST and delivery of HTS through community‐based hubs. When men are reached, they are willing to test for HIV. Reaching men thus requires implementers to adapt their HTS delivery strategies to meet men's needs.

**Clinical Trial Number:**

NCT01900977

## 1 INTRODUCTION

Data from sub‐Saharan African countries consistently show that men are less likely than women to test for HIV [1, 2, 3]. In Zambia, the 2013–14 Demographic and Health Survey (DHS) showed that 37% of men aged 15–49 reported testing for HIV in the last 12 months compared to 46% of women [4, 5]. Despite an increase in HIV testing coverage reported in the 2018 Zambian DHS, with 52% of men and 64% of women reporting testing in the last 12 months [6], efforts to increase HIV testing services (HTS) uptake among men are needed.

Community‐based delivery of HTS, which includes home‐based, workplace, campaign‐style and mobile‐based delivery of HTS, is a strategy to reach men missed by facility‐based HTS [7]. In Zimbabwe, Uganda and Kenya, mobile‐ and campaign‐style HTS reached more men than facility‐based testing; however, a gap in coverage remained [8, 9, 10]. While it is acknowledged that a combination of different community‐based strategies is needed, knowledge on what combinations work, for whom and to what extent they work to reach men with HTS is limited [11]. In Uganda and Kenya, the Sustainable East Africa Research in Community Health (SEARCH) Trial used a campaign‐style multiple disease‐based strategy followed by home‐based strategy. This combination resulted in high coverage, yet a lower proportion of men tested for HIV compared to women (86% vs. 92%, respectively) [12].

From an early stage, data from the HIV Prevention Trials Network (HPTN) 071(PopART) community‐randomized trial conducted in Zambia and South Africa showed that fewer men than women knew their HIV status, primarily because men were less likely to be found at home by community health workers (CHWs) conducting door‐to‐door visits [13]. Therefore, additional strategies were implemented to reach men during the PopART intervention, including campaign‐style and mobile‐based delivery of HTS. In this analysis, we present the relative contribution of these strategies as well as the impact of community hubs offering HTS, which were implemented after completion of the HPTN‐071 (PopART) trial, on reaching men with HTS.

## 2 METHODS

### 2.1 The HPTN 071 (PopART) trial

**Figure 1:**
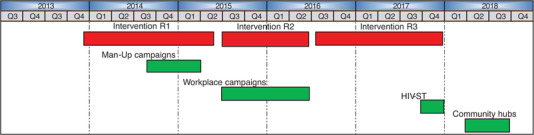
Timeline of PopART intervention rounds and complementary strategies to reach men. Abbreviations: HIVST, HIV self‐testing; PopART, Population Effects of Antiretroviral Therapy to Reduce HIV Transmission.

The HPTN 071 (PopART) trial was a three‐arm community‐randomized trial that evaluated the impact of a combination HIV prevention package, including universal testing‐and‐treatment, on HIV incidence in 21 communities in Zambia and South Africa. Details of the trial design are described elsewhere [14]. From December 2013 to December 2017, three intervention rounds (IR1, IR2 and IR3) of door‐to‐door service delivery were conducted in all eight PopART intervention communities in Zambia (Figure 1). The intervention was delivered by a pair of trained CHWs covering a geographical zone of ∼450 households. The PopART intervention consisted of an offer of home‐based HTS and referral for immediate antiretroviral therapy initiation, screening and referral for tuberculosis and sexually transmitted infections, referral for voluntary medical male circumcision (VMMC) and condom provision. All referrals were made to the local governmental health facilities. Within each IR, repeated household visits were conducted to try to contact *all* household members. The actual number of repeated visits was at the discretion of the CHW.

### 2.2 Development of additional HTS delivery strategies

A needs assessments through one focus group discussion with men in Lusaka, eight meetings with community advisory board (CABs) members and feedback from PopART field staff during routine staff meetings were conducted to identify barriers to accessing care. Guidelines were developed to ensure uniformity in implementation across communities.

Step one was mapping local socio‐economic places per community, including markets, factories, churches and informal workplaces, such as gardening and brewing areas.

Step two was engaging all relevant stakeholders, including CABs, neighbourhood health committees and other local leadership. The last step was community sensitization through drama and mega‐phone, a week prior to HTS strategy delivery.

#### 2.2.1 “Man‐up” campaigns

From September 2014 to April 2015, “Man‐up” campaigns, consisting of community‐wide 2‐day health fairs held in open areas (e.g. football pitch), were organized in five of the eight PopART communities. Staff partnered with local healthcare providers to offer healthcare services, including blood pressure measurement, diabetes, prostate cancer screening, eye testing and on site provision of VMMC. Each service had its own designated tent. A tent labelled “PopART” offered HTS alongside the full PopART package as offered at a household. A raffle for male attendees was included to encourage participation.

#### 2.2.2 Workplaces and social spaces

From June 2015 to June 2016, HTS campaigns in formal (e.g. factories)/informal (e.g. markets) workplaces and social spaces (e.g. churches and football matches) were conducted in all eight PopART communities.

#### 2.2.3 Revisits with HIV self‐testing

During the last 4 months of the PopART intervention (September–December 2017), CHWs revisited households in all eight communities where one or more household members were previously absent or declined HTS. Analyses were restricted to four communities that were not part of a previous nested trial of HIV self‐testing (HIVST) [15]. Upon visiting these households, HIVST, including for secondary distribution, was offered with participants choosing between finger‐prick testing or HIVST.

### 2.3 Community‐based hubs

From April to October 2018, after completion of the PopART intervention, community “hubs” delivering the PopART package were established in all eight communities. Hubs were located in high‐density areas using tents at markets, church/school grounds, in shops or community halls. The locations of the hubs were determined through consultative meetings with CABs, local Ministry of Health representatives and PopART staff. The hubs were static, though some shifted based on community response and attendance. In the hubs, services were offered by a pair of CHWs to people self‐reporting to the hub. Each hub served a population of ∼10,000 people.

HIV testing for all abovementioned strategies followed the Zambia national testing algorithm. For finger‐prick testing, Determine® (Alere) was used as a screening test, with Unigold® (Unitech Biotech Ltd) as a confirmatory test. For HIVST, Oraquick® HIVST kit was used as the screening test, with Determine® and Unigold® in parallel as confirmatory tests. All participants diagnosed HIV positive were referred to the health facility for care and treatment. Individuals self‐reporting their HIV‐positive status were not offered HTS by CHWs.

### 2.4 Data collection

#### 2.4.1 PopART intervention

At the first household visit (IR1), CHW enumerated all household members, including absent members, and collected data on history of HIV testing and PopART service uptake from household members consenting to participate during the household visit. CHWs used handheld electronic data capture (EDC) devices for data collection [13]. At IR2 and IR3, enumeration was updated and adjusted for in‐ and out‐migration.

#### 2.4.2 “Man‐up” campaigns

For every campaign attendee, name, age and sex were used to identify the client and to access their household enumeration records in the central database. If the client had not been enumerated, enumeration was conducted. Data were recorded in the same way as door‐to‐door data collection. If the client was from outside the PopART intervention area or had already participated in the PopART intervention, they were offered all PopART services but data were not collected or recorded.

#### 2.4.3 Workplaces and social spaces

Data were collected on specially designed paper data collection tools, identical to EDC tools used during door‐to‐door visits. This enabled CHWs to identify previously recorded electronic data and reconcile newly obtained data during campaigns with existing electronic data.

#### 2.4.4 Revisits with HIVST

We used the standard PopART intervention EDC, with additional data recorded for HIVST as described elsewhere [15].

#### 2.4.5 Community‐based hubs

Using EDCs, CHWs entered one record per participant visit with name, age, sex, telephone number, self‐reported HIV status and uptake of HTS. These data were not linked to data collected during the HPTN 071 (PopART) trial. During the last 3.5 months of service delivery, CHW recorded the answer to two additional questions: “how long the client lived in the community” and, if >1 year, “whether client ever met a CHW during the door‐to‐door PopART intervention.”

### 2.5 Data analysis

All analyses were restricted to men aged ≥18 years at the time of first enumeration and first consent to participate. To estimate the proportion reached with HTS overall during 4 years of PopART intervention, we merged data for IR1, IR2 and IR3 for the denominator. Participants repeatedly enumerated, consented or tested across different rounds, counted as one observation.

To illustrate the compound effect of multiple IRs, we calculated the proportion participating and testing among those enumerated more than once across three rounds compared to those enumerated once across multiple rounds.

To compare participation between IRs, for each IR we reported the total number of men enumerated (as the denominator), self‐reported HIV positive, eligible for testing (not self‐reported HIV positive), uptake of HTS and testing HIV positive. These estimates combined data from door‐to‐door service delivery, “Man Up” campaigns, workplaces, social places and revisits with HIVST.

We then reported the same measures for each IR, but restricted to communities that implemented complementary strategies, and calculated the relative contribution of each complementary strategy to the percentage of men reached with HTS.

#### 2.5.1 Community hubs

Repeated visits by the same individual were identified by generating a unique identifier based on community, name, sex and either telephone number or age. These were removed, leaving one observation per participant with information on ever‐tested for HIV and test result.

We first compared the age, community of residence, uptake of HTS and self‐reported HIV‐positive status of men visiting hubs during the first 3.5 months of implementation with those visiting during the last 3.5 months. Next, we estimated the overall number of men HIV testing at the hubs who had lived in the community during the PopART intervention but had not seen a CHW. For this, we used, as the denominator, the number of men enumerated by CHWs in IR3 but who never participated in the PopART intervention, either because they were absent during household visits or declined participation. For the numerator, we added up two components. First, we calculated the number of men visiting hubs during the *last* 3.5 months who said that they had been living in the community for >1‐year and had never met a CHW. For this group of participants, we fit a random effects logistic regression model with “living in the community for >1‐year” or “living in the community for >1‐year and not having met a CHW” as the dependent variable, community of residence and age group (18–19, 20–24, 25–54 and ≥55 years) as explanatory variables and hub as the random effect. We assessed the goodness‐of‐fit of the regression model (which assumed that the pattern by age group in the proportion with the outcome was the same in each community) by fitting a linear regression model of the observed proportion with the outcome (y‐axis) on the predicted proportion with the outcome (x‐axis) for each combination of community and age group (32 combinations). Using this linear regression model, we compared the slope and intercept of the regression line with the 1:1 (y = x) line, and estimated the *R* ^2^ value. The parameters of this model were then used to estimate the probability of “living in the community for >1‐year” and “living in the community for >1‐year and not having seen a CHW” for participants that visited hubs in the *first* 3.5 months of implementation. Finally, to estimate the number of men HIV testing at the hubs who had not been seen by a CHW despite residing in the community, we combined the data for the two time periods (actual number from *last 3.5 months* with estimated number from *first 3.5 months*).

### 2.6 Ethical considerations

During all activities, participants were asked for verbal informed consent. Those agreeing to an HIV test provided written consent according to national standards. The study and above procedures were approved by the ethics committees of the London School of Hygiene & Tropical Medicine (Ref: 6326) and the University of Zambia (REF: 011‐11‐12). For hubs, verbal consent was obtained for accessing HTS and data collection. All processes were approved by the Zambian Ministry of Health.

## 3 RESULTS

### 3.1 PopART Intervention

**Figure 2:**
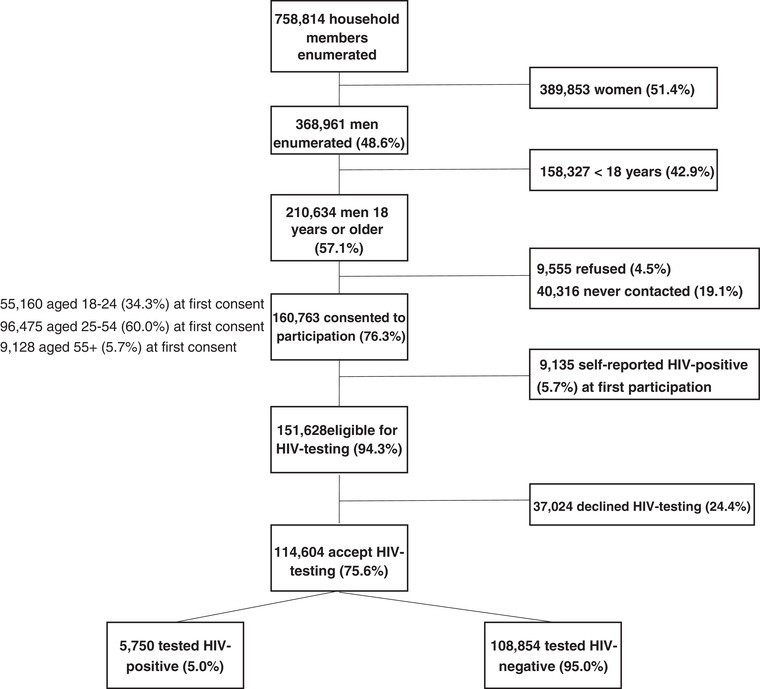
Total number of men ever enumerated, consented at least once to participation and tested for HIV at least once after 4 years (three annual rounds) of PopART intervention (Zambia 2013–2017). Abbreviation: PopART, Population Effects of Antiretroviral Therapy to Reduce HIV Transmission.

Over 4 years of the PopART intervention, 758,814 household members were enumerated in the eight intervention communities at least once. Of these, 48.6% were men. Of men enumerated, 57.1% were ≥18 years and 76.3% consented to participate. Overall, 151,628 were eligible for HTS and 75.6% accepted HTS at least once, of whom 5.0% tested HIV positive (Figure 2).

The proportion of men enumerated more than once across IRs was 43.4% (91,454/210,634), among whom 86.0% (78,673) consented at least once and were eligible for testing. Among these, 74.0% (58,244/78,673) tested for HIV. The proportion of men enumerated only once across the IRs was 56.6% (119,180/210,634). 68.9% (82,090) consented and were eligible for testing, of these 68.7% (56,360/82,090) tested for HIV.

#### 3.1.1 Intervention round one

In IR1, among men aged ≥18 years who were enumerated, 76.8% were reached by the door‐to‐door service and complementary strategy. Excluding 5168 self‐reported HIV positive, 62.5% tested for HIV. The yield of HIV‐positive test results was 5.2% (Table 1).

**Table 1:** Men 18 years and older contacted at the end of each round of the HTPN 071 (PopART) intervention in all eight communities in Zambia showing the combined effect of door‐to‐door and complementary strategies

	Intervention round 1	Intervention round 2	Intervention round 3
	(12/2013–05/2015)	(06/2015–07/2016)	(08/2016–12/2017)
Total men enumerated	122,572	109,042	113,792
Overall outcomes			
Men reached (% of enumerated) by the end of round ‐ refused ‐ not contacted	**94,028/122,572 (76.8%)** ‐ 9886/122,572 (8.0%) ‐ 18,658/122,572 (15.2%)	**70,304/109,042 (64.5%)** ‐ 3803/109,042 (3.5%) ‐ 34,935/109,042 (32.0%)	**73,579/113,792 (64.7%)** ‐ 2732/113,792 (2.4%) ‐ 37,481/113,792 (32.9%)
Self‐reported HIV positive	5168/94,028 **(5.5%)**	5478/70,304 **(7.8%)**	5886/73,579 **(8.0%)**
Tested for HIV (among those eligible, i.e. not self‐reported HIV positive)	55,568/88,860 **(62.5%)**	42,799/64,826 **(66.0%)**	54,235/67,693 **(80.1%)**
Tested HIV positive	2908/55,568 **(5.2%)**	1424/42,799 **(3.3%)**	1426/54,235 **(2.6%)**
Know HIV status (self‐reported +, tested by PopART)	60,736/94,028 **(64.6%)**	48,277/70,304 **(66.7%)**	60,121/73,579 **(81.7%)**

Abbreviation: PopART, Population Effects of Antiretroviral Therapy to Reduce HIV Transmission.

For the “Man Up” campaign, 63% of attendees were men. Of those, 2261/2905 (77.8%) lived in the intervention area and accessed PopART services, among whom 982 (43.4%) had not previously participated in door‐to‐door services. This accounts for 1.4% additional men being reached; overall, 82.7% men tested for HIV with 3.1% (25/810) testing HIV positive (Table 2). Screening for hypertension and diabetes was the most frequently accessed “non‐PopART” services (data not shown).

**Table 2:** Men 18 years and older contacted by door‐to‐door and complementary strategies implemented during three rounds of the HTPN 071 (PopART) intervention, restricted to communities where complementary strategies were implemented (excluding community‐based hubs)

	Intervention round 1	Intervention round 2	Intervention round 3
	(12/2013–05/2015)	(06/2015–07/2016)	(08/2016–12/2017)
Door‐to door			
Scope and scale	Universal coverage in 5/8 communities	Universal coverage in 8/8 communities	Universal coverage in 4/8 communities
Men reached	40,894/55,923 **(73.1%)**	69,073/109, 042 **(63.3%)**	33,847/74,128 **(45.7%)**
Self‐reported HIV positive	2279/40,894 **(5.6%)**	5478/69,073 **(7.9%)**	3028/33,847 **(8.9%)**
Tested for HIV	25,344/38,615 **(65.6%)**	41,568/63,595 **(65.4%)**	23,847/30,819 **(77.4%)**
Tested HIV positive	1371/25,344 **(5.4%)**	1302/41,568 **(3.1%)**	728/23,847 **(3.1%)**
Additional activities	“Man Up” campaign	Local workplaces, social gathering places and extended hours	**Re‐visit of households including the offer of HIV self‐testing (HIVST)^b^**
Scope and scale	5/8 communities 1 weekend campaign per community	8/8 communities different strategies depending on local gathering or work places	4/8 communities targeted revisits to households with unreached or untested men and households not visited yet, with the addition of HIVST (3 months)
Number reached who had not been seen in door‐to‐door (% of enumerated)	982/55,923 **(1.4%)**	1231/109,042 **(1.1%)**	11,548/74,128 **(15.6%)**
Self‐reported HIV positive (% of those reached)	–	–	720/11,548 **(6.2%)**
Tested for HIV	810^a^/982 (82.5%)	1231 (100%)	8353/10,828 **(77.1%)**
Tested HIV positive	25/810 (3.1%)	122/1231 **(9.9%)**	180/8353 **(2.2%)**
Overall outcomes			
Men reached (% of enumerated) by the end of round ‐refused ‐not contacted	**41,876/55,923 (74.9%)** ‐4097/55,923 (7.3%) ‐9950/55,923 (17.8%)	**70,304/109,042 (64.5%)** ‐3803/109,042 (3.5%) ‐34,935/109,042 (32.0%)	**45,395/74,128 (61.2%)** ‐1937/74,128 (2.6%) ‐26,796/74,128 (36.2%)
Self‐reported HIV positive	2279/41,876 **(5.4%)**	5478/70,304 **(7.8%)**	3748/45,395 **(8.3%)**
Tested for HIV (among those eligible)	26,154/39,597 **(66.1%)**	42,799/64,826 **(66.0%)**	32,200/41,647 **(77.3.0%)**
Tested HIV positive	1396/26,154 **(5.3%)**	1424/42,799 **(3.3%)**	908/32,200 **(2.8%)**
Know HIV status (self‐reported +, tested by PopART)	28,433/41,876 **(67.9%)**		35,948/45,395 **(79.2%)**

Abbreviation: PopART, Population Effects of Antiretroviral Therapy to Reduce HIV Transmission.

^a^ Included participants who visited the campaign but were seen and tested before in the household.

^b^ Included only the four communities that did not participate in the nested HIVST trial [12].

#### 3.1.2 Intervention round two

In IR2, door‐to‐door and complementary strategies reached 64.5%. Excluding 5478 who self‐reported being HIV positive, 66.0% men tested for HIV. The HIV‐positive yield was 3.3% (Table 1). Workplace and social places HTS reached an additional 1.1% men. The yield of HIV‐positive results for the complementary testing was 9.9% (Table 2). Workplace HTS reached more men compared to social places HTS (data not shown).

#### 3.1.3 Intervention round three

In IR3, among men enumerated, 64.7% were reached with door‐to‐door revisits with the option to HIVST. The percentage of men tested for HIV, excluding 5886 who self‐reported being HIV positive, increased, with 80.1% accepting the offer of HTS. The HIV‐positive yield was 2.6% (Table 1).

Among 74,128 men living in 4/8 communities, during revisits with HIVST, 15.6% were reached. The yield of HIV‐positive test results was 2.2% (Table 2).

### 3.2 Community hubs

**Figure 3:**
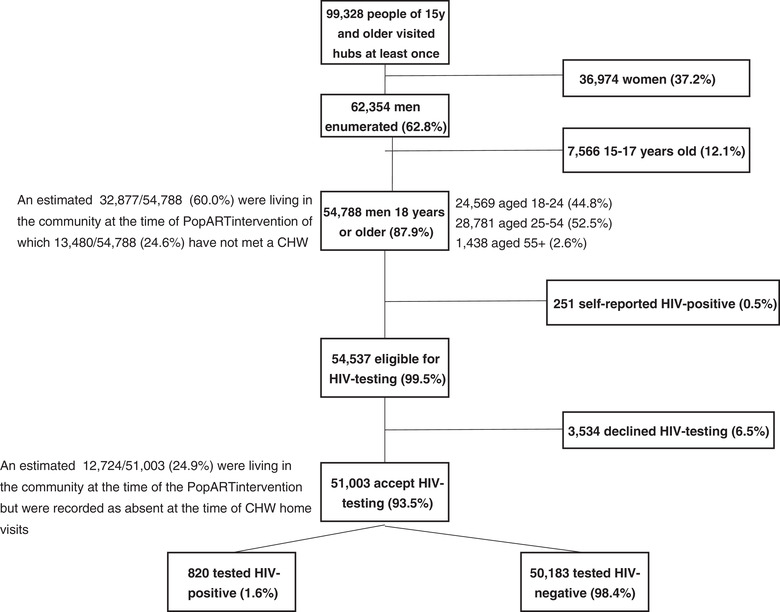
Total number of men attending hubs (Zambia, April 2018–October 2018). Abbreviations: CHW, community health worker; PopART, Population Effects of Antiretroviral Therapy to Reduce HIV Transmission.

During 7 months of implementation, 99,328 individuals aged ≥15 visited hubs at least once (Figure 3); 62.8% were male. The percentage of men aged ≥18 years attending hubs was equivalent to 48.1% (54,788/113,792) of the total population of men enumerated in IR3. 44.8% were aged 18–24. Of those eligible, 93.5% accepted HIV testing. Fifty‐eight percent (29,490/51,003) chose HIVST. 1.6% tested HIV positive.

Among the 31,506 men visiting hubs during the first 3.5 months, 15.1% were aged 18–19 years, 29.2% 20–24 years, 52.9% 25–54 years and 2.8% ≥55 years. This was similar among 23,282 men who visited hubs in the second 3.5 months (16.0%, 29.5%, 52.1% and 2.4%, respectively). The uptake of HTS among those eligible (91.6% versus 96.1%, respectively), proportion of self‐reported HIV positive (0.5% versus 0.4%, respectively) and proportion of people coming forward from different communities (data not shown) were similar.

Of the 113,792 men enumerated in IR3, 32,877 (28.9%) who lived in the PopART communities at the time of the intervention were reached by the hubs. Excluding 158 (0.5%) self‐reported HIV‐positive men, 30,759 (94.0%) tested for HIV. The yield of HIV‐positive results was 1.7%. Of the 37,481 men enumerated in IR3 but not contacted (Table 1), 36.0% were reached in hubs. Among those eligible, 94.8% tested for HIV. The yield of HIV‐positive results was 2.2% (Table 3).

**Table 3:** Description of men aged 18 and older contacted at community hubs in relation to men contacted and not contacted during IR3 of the HTPN 071 (PopART) intervention in eight communities in Zambia

PopART intervention round 3	
Men reached (% of enumerated) by end of IR3 ‐refused ‐not contacted	**73,579/113,792 (64.7%)** ‐2732/113,792 (2.4%) ‐37,481/113,792 (32.9%)
Self‐reported HIV positive	5886/73,679 **(8.9%)**
Tested for HIV (among those eligible, i.e. not self‐reported HIV positive)	54,235/67,793 **(80.0%)**
Tested HIV positive	1426/54,235 **(2.6%)**
Know HIV status (self‐reported +, tested by PopART)	60,121/73,579 **(81.7%)**
Men aged 18 years and older attending hubs overall	
Men reached (among total enumerated at the end of intervention round 3)	**54,788/113,792 (48.1%)**
Self‐reported HIV positive	251/54, 788 **(0.5%)**
Tested for HIV (among those eligible, i.e. not self‐reported HIV positive)	51,003/54,537 **(93.5%)**
Tested HIV positive	820/51,003 **(1.6%)**
Know HIV status (self‐reported + tested at hub)	51,254/54,788 **(94.0%)**
Men aged 18 years and older attending hubs and lived in the community at the time of the PopART intervention	
Men reached (among total enumerated at the end of intervention round 3)	**32,877/113,792 (28.9%)**
Self‐reported HIV positive	158/32,877 **(0.5%)**
Tested for HIV (among those eligible, i.e. not self‐reported HIV positive)	30,759/32,719 **(94.0%)**
Tested HIV positive	510/30,759 **(1.7%)**
Know HIV status (self‐reported + tested at hub)	30,917/32,877 **(93.5%)**
Men aged 18 years and older attending hubs, lived in the community at the time of the PopART intervention but never met a CHW	
Men reached (among total recorded as absent at the end of intervention round 3)	**13,480/37,481 (36.0%)**
Self‐reported HIV positive	52/13,480 **(0.4%)**
Tested for HIV (among those eligible, i.e. not self‐reported HIV positive)	12,724/13,428 **(94.8%)**
Tested HIV positive	275/12,724 **(2.2%)**
Know HIV status (self‐reported + tested at hub)	12,776/13,480 **(94.8%)**

Abbreviations: CHW, community health worker; PopART, Population Effects of Antiretroviral Therapy to Reduce HIV Transmission.

Of men enumerated in IR3, 64.7% (73,579/113,792) were reached prior to hub implementation. We estimated that an additional 13,480 men were reached by hubs, for an overall estimate of 76.5% (87,059/113,792) reached.

The linear regression model had a slope of 0.95 (95% CI 0.94–0.96) and an *R* ^2^ value of 99.6%. The corresponding values from the linear regression model for the proportion of men “living in the community for more than one year and not having seen a CHW” had a slope of 1.06 (95% CI 1.03–1.09) and an *R* ^2^ value of 98.4%.

## 4 DISCUSSION

The PopART intervention reached a high proportion of men (∼70%) with HTS via a CHW‐led door‐to‐door approach. To reach the remaining 30%, additional strategies were implemented with variable success. HTS delivered through community hubs were a valuable complement to door‐to‐door services providing HTS to men who were not at home during household visits. Accessing hub services appeared to be more acceptable and appealing to young men than accessing services at home.

Evidence from systematic reviews suggest that mobile and campaign style HTS reach a higher proportion of men compared to home‐based delivery of HTS [8, 16, 17]. The SEARCH Trial used a hybrid testing strategy with campaign style testing 64% of men, followed by home‐based HTS testing 22% of men [12]. This contrasts with our findings that home‐based delivery reached the highest proportion of men, and that mobile and campaign style strategies were not sufficient to find men missed by home‐based HTS. This contrast could be explained firstly because, PopART home‐based testing was offered *before* campaign style HTS. Secondly, the PopART intervention was intensive, with CHWs making repeated household visits. In addition, we evaluated complementary community HTS strategies in parallel or after intensive door‐to‐door HTS; the potential coverage of standalone workplace‐ or campaign‐like HTS or HTS through community hubs cannot be evaluated. This limits the generalizability of our findings that campaign‐like HTS have a low impact among men.

In parallel to intensive door‐to‐door HTS, revisits to households with HIVST reached more additional men than campaign or workplace strategies. This corroborates previous findings on increased knowledge of HIV status following the offer of HIVST [15].

Community‐based hubs were popular among men and reached a high proportion (36%) of men missed during the PopART intervention, increasing the proportion of men reached in IR3 from 64.7% to 76.5%. The high uptake of HTS at community hubs by community members who never met a CHW *even* after 4 years of intensive door‐to‐door HTS suggests that a combination of door‐to‐door and hub‐based strategies is complementary and can reach different sub‐groups of men in high HIV‐prevalence settings. The lower HIV positivity among men testing at hubs who never tested at home by the door‐to‐door PopART intervention compared with the men who tested at hubs and had previously tested at home during door‐to‐door activities also indicates that hubs can reach different sub‐groups of men (hub attendants were generally younger than men reached at home). The impact of door‐to‐door testing may be high because of repeated visits increasing the likelihood of reaching men and/or because a comprehensive package of health services was offered. While door‐to‐door testing is often seen as a high‐cost unsustainable strategy, it is less costly than expected, with a mean cost of $6.53 per person year of delivery in Zambia [18]. Costs can be reduced by delivering door‐to‐door testing in different formats and intensities. Analysis of uptake of HTS showed that among men who tested at any time during IR1, 52.7% tested at the first visit [19]. While hubs cost‐effectiveness was not evaluated, fewer staff were required than door‐to‐door testing while covering the same geographical area, thus reasonable to assume lower implementation costs. Additionally, the hubs were flexible in terms of location. We believe this would increase the feasibility and sustainability of hubs.

Qualitative data suggested that the high uptake of HTS in hubs was due to convenience of delivery, especially for young mobile men in informal employment. Hubs were located in places with high volumes of people walking by. Barriers to men's uptake of HTS include factors, such as men's mobility due to livelihood demands and costs of accessing HTS resulting in loss of income [10, 12, 20]. These barriers also emerged through our qualitative work and was similar to other mobile HTS study findings [21, 22]. Although our hubs were only implemented for 7 months and reached 54,788 men compared to 73,579 men in 12–16 months of the main PopART intervention, had they been available for a longer period, they may have reached a higher number of men missed during door‐to‐door HTS [20]. For low‐resource settings, we recommend a less‐intensive CHW‐led door‐to‐door strategy with one household visit followed by or in parallel with the offer of HTS via community hubs to reach men.

In the context of the COVID‐19 epidemic, access to HTS is more important than ever, considering less‐ than‐usual access to facility‐based HTS [18]. COVID‐19 prevention strategies, including vaccination, can be integrated with door‐to‐door HTS (with HIVST) and community hubs to contribute to both HIV and COVID‐19 epidemic control.

### 4.1 Strengths and limitations

The strength of our research is that we enumerated all men in our community providing us with a denominator to measure the impact of HTS strategies at population level.

The limitations of our research are: for additional strategies, we report on those who had not yet been reached door‐to‐door, but may have eventually been reached with door‐to‐door HTS; our data on HIVST do not allow us to differentiate the relative contribution of revisiting households versus the addition of HIVST to the prevention package; and community hubs were established *after* door‐to‐door HTS. Data collected at hubs could not be linked at individual level to data collected during the PopART intervention. Lastly, data collection on previous contact with CHWs was only introduced halfway in the implementation period and was based on self‐report, which could have led to over‐ or underreporting. To estimate the proportion of men visiting hubs among those who were missed by the PopART intervention, we have made the strong assumption that those visiting hubs in the second 3.5 months were similar to the group visiting hubs in the first 3.5 months. However, we are confident, reliable projections were made given the similarity of the two groups in terms of age, community of residence and uptake of testing, and given the goodness‐of‐fit of the regression model used for projections.

## 5 CONCLUSIONS

Men are often considered “hard to reach” with HTS [23] resulting in men less likely to know their HIV status compared to women in sub‐Saharan Africa. Using a combination of universal home‐based HTS and community hubs, we found that once men were reached, the uptake of HTS was high. In real‐life settings, we recommend a CHW‐led door‐to‐door strategy with a minimum of one household visit followed by or in parallel to community‐based hubs to reach men. HTS programs and policies need to recognize that men are not “hard to reach” but rather that HTS may be “hard to reach” and need to be adapted to be responsive to men's needs. If HTS is provided in more acceptable, convenient and accessible ways for different groups of men, high uptake can be achieved.

## COMPETING INTERESTS

The authors declare no competing interests.

## AUTHORS’ CONTRIBUTIONS

MP and AS conceptualized the manuscript. AS conducted the analysis. MP, AS, MS, BC and CM oversaw in‐country data collection. MP and AS led the manuscript writing. BH, MS, MS, VB, SF, KS, RH and HA were involved in drafting the manuscript and provided critical feedback on the full manuscript. All authors read and approved the final manuscript.

## FUNDING

HPTN 071 was sponsored by the National Institute of Allergy and Infectious Diseases (NIAID) under Cooperative Agreements UM1‐AI068619, UM1‐AI068617 and UM1‐AI068613, with funding from the U.S. President's Emergency Plan for AIDS Relief (PEPFAR). Additional funding is provided by the International Initiative for Impact Evaluation (3ie) with support from the Bill & Melinda Gates Foundation, as well as by NIAID, the National Institute on Drug Abuse (NIDA) and the National Institute of Mental Health (NIMH), all part of NIH.

## DISCLAIMER

The content is solely the responsibility of the authors and does not necessarily represent the official views of the NIAID, NIMH, NIDA, PEPFAR, 3ie or the Bill & Melinda Gates Foundation.

## References

[1] Staveteig S , Wang S , Head SK , Bradley SEK , Nybro E . Demographic patterns of HIV testing uptake in sub‐Saharan Africa. DHS Comparative Reports. Calverton, MD; 2013.

[2] UNAIDS . Blind spot: reaching out to men and boys. 2017 [cited 2019 Sep 9]. Available from: https://www.unaids.org/sites/default/files/media_asset/blind_spot_en.pdf

[3] Shand T , Thomson‐de Boor H , van den Berg W , Peacock D , Pascoe L . The HIV blind spot: men and HIV testing, treatment and care in sub‐Saharan Africa. IDS Bull. 2014;45(1):53–60.

[4] Ministry of Health Zambia . Zambia Population‐based HIV Impact Assessment (ZAMPHIA) 2016: final report. 2019 [cited 2019 Sep 19]. Available from: https://phia.icap.columbia.edu/wp‐content/uploads/2019/02/ZAMPHIA‐Final‐Report__2.22.19.pdf

[5] Central Statistical Office (CSO) [Zambia] , Ministry of Health (MOH) [Zambia] and II . Zambia Demographic Health Survey 2013–14. 2014 [cited 2019 Sep 9]. Available from: https://www.dhsprogram.com/pubs/pdf/fr304/fr304.pdf

[6] CSO , CBoH , ORC Macro . The DHS Program ‐ Zambia: DHS, 2018 ‐ final report (English). [cited 2021 Aug 20]. Available from: https://dhsprogram.com/publications/publication‐fr361‐dhs‐final‐reports.cfm

[7] Sharma M , Ying R , Tarr G , Barnabas R . Systematic review and meta‐analysis of community and facility‐based HIV testing to address linkage to care gaps in sub‐Saharan Africa. Nature. 2015;528(7580):S77–85. 26633769 10.1038/nature16044 PMC4778960

[8] Sharma M , Barnabas RV , Celum C . Community‐based strategies to strengthen men's engagement in the HIV care cascade in sub‐Saharan Africa. PLoS Med. 2017;14(4):e1002262. 28399122 10.1371/journal.pmed.1002262 PMC5388461

[9] Hensen B , Taoka S , Lewis JJ , Weiss HA , Hargreaves J . Systematic review of strategies to increase men's HIV‐testing in sub‐Saharan Africa. AIDS. 2014;28(14):2133–45. 25062091 10.1097/QAD.0000000000000395 PMC4819892

[10] Camlin CS , Ssemmondo E , Chamie G , El Ayadi AM , Kwarisiima D , Sang N , et al. Men “missing” from population‐based HIV testing: insights from qualitative research. AIDS Care. 2016;28(sup3):67–73. 27421053 10.1080/09540121.2016.1164806 PMC5749410

[11] Colvin CJ . Strategies for engaging men in HIV services. Lancet HIV. 2019;6(3):e191–200. 30777726 10.1016/S2352-3018(19)30032-3

[12] Chamie G , Clark TD , Kabami J , Kadede K , Ssemmondo E , Steinfeld R , et al. A hybrid mobile approach for population‐wide HIV testing in rural east Africa: an observational study. Lancet HIV. 2016;3(3):e111–9. 26939734 10.1016/S2352-3018(15)00251-9 PMC4780220

[13] Hayes R , Floyd S , Schaap A , Shanaube K , Bock P , Sabapathy K , et al. A universal testing and treatment intervention to improve HIV control: one‐year results from intervention communities in Zambia in the HPTN 071 (PopART) cluster‐randomised trial. PLoS Med. 2017;14(5):e1002292. 28464041 10.1371/journal.pmed.1002292 PMC5412988

[14] Hayes R , Ayles H , Beyers N , Sabapathy K , Floyd S , Shanaube K , et al. HPTN 071 (PopART): rationale and design of a cluster‐randomised trial of the population impact of an HIV combination prevention intervention including universal testing and treatment – a study protocol for a cluster randomised trial. Trials. 2014;15(1):57. 24524229 10.1186/1745-6215-15-57 PMC3929317

[15] Mulubwa C , Hensen B , Phiri MM , Shanaube K , Schaap AJ , Floyd S , et al. Community based distribution of oral HIV self‐testing kits in Zambia: a cluster‐randomised trial nested in four HPTN 071 (PopART) intervention communities. Lancet HIV. 2019;6(2):e81–92. 30584047 10.1016/S2352-3018(18)30258-3 PMC6361868

[16] Sweat M , Fonner V . Overcoming resistance to HIV testing in sub‐Saharan Africa. Lancet HIV. 2016;3(3):e106–7. 26939730 10.1016/S2352-3018(16)00004-7

[17] Asaolu IO , Gunn JK , Center KE , Koss MP , Iwelunmor JI , Ehiri JE . Predictors of HIV testing among youth in sub‐Saharan Africa: a cross‐sectional study. PLoS One. 2016;11(10):e0164052. 27706252 10.1371/journal.pone.0164052 PMC5051677

[18] Thomas R , Probert WJM , Sauter R , Mwenge L , Singh S , Kanema S , et al. Cost and cost‐effectiveness of a universal HIV testing and treatment intervention in Zambia and South Africa: evidence and projections from the HPTN 071 (PopART) trial. Lancet Glob Health. 2021;9(5):e668–80. 33721566 10.1016/S2214-109X(21)00034-6 PMC8050197

[19] Shanaube K , Schaap A , Floyd S , Phiri M , Griffith S , Chaila J , et al. What works — reaching universal HIV testing: lessons from HPTN 071 (PopART) trial in Zambia. AIDS. 2017;31(11):1555–64. 28471766 10.1097/QAD.0000000000001514 PMC5491236

[20] Musheke M , Ntalasha H , Gari S , Mckenzie O , Bond V , Martin‐Hilber A , et al. A systematic review of qualitative findings on factors enabling and deterring uptake of HIV testing in sub‐Saharan Africa. BMC Public Health. 2013;13(1):220. 23497196 10.1186/1471-2458-13-220 PMC3610106

[21] van Rooyen H , McGrath N , Chirowodza A , Joseph P , Fiamma A , Gray G , et al. Mobile VCT: reaching men and young people in urban and rural South African pilot studies (NIMH Project Accept, HPTN 043). AIDS Behav. 2013;17(9):2946–53. 23142856 10.1007/s10461-012-0368-x PMC3597746

[22] Sweat M , Morin S , Celentano D , Mulawa M , Singh B , Mbwambo J , et al. Community‐based intervention to increase HIV testing and case detection in people aged 16–32 years in Tanzania, Zimbabwe, and Thailand (NIMH Project Accept, HPTN 043): a randomised study. Lancet Infect Dis. 2011; 11(7):525–32. 21546309 10.1016/S1473-3099(11)70060-3 PMC3156626

[23] Staveteig S , Croft TN , Kampa KT , Head SK . Reaching the ‘first 90’: gaps in coverage of HIV testing among people living with HIV in 16 African countries. PLoS One. 2017;12(10):1–16. 10.1371/journal.pone.0186316 PMC5638499 29023510

